# The prognostic value of lncRNA SNHG1 in cancer patients: a meta-analysis

**DOI:** 10.1186/s12885-019-5987-4

**Published:** 2019-08-07

**Authors:** Bingzi Dong, Xian Chen, Yunyuan Zhang, Chengzhan Zhu, Qian Dong

**Affiliations:** 1 Department of Endocrinology and Metabolism, The Affiliated Hospital of Qingdao University, Qingdao, 266003 China; 2 Department of Clinical Laboratory, The Affiliated Hospital of Qingdao University, Qingdao, 266003 China; 3 Department of Hepatobiliary and Pancreatic Surgery, The Affiliated Hospital of Qingdao University, Qingdao, 266003 China; 4 Department of Pediatric Surgery, The Affiliated Hospital of Qingdao University, Qingdao, 266003 China; 5 Shandong Key Laboratory of Digital Medicine and Computer Assisted Surgery, The Affiliated Hospital of QingDao University, Qingdao, 266003 China

**Keywords:** Cancer, Prognosis, Long non-coding RNA, *SNHG1*

## Abstract

**Background:**

Increasing evidence revealed that high expression level of lncRNA *SNHG1* was associated with the unfavorable prognosis of cancer and maybe used as a valuable biomarker for cancer patients. The present meta->analysis is to analyze existing data to reveal potential clinical application of *SNHG1* on cancer prognosis and tumor progression. All of the included studies were collected through a variety of retrieval strategies. And the articles were qualified by MOOSE and PRISMA checklists.

**Methods:**

Up to Mar 20, 2018, literature collection was performed by comprehensive search through electronic databases, including the Cochrane library, PubMed, Embase, Web of science, Springer, Science direct, and three Chinese databases: CNKI, Weipu, and Wanfang. We analyzed 14 studies that met the criteria, and concluded that the increased *SHNG1* level was correlated with poor OS and tumor progression.

**Results:**

The combined results indicated that elevated *SNHG1* expression level was significantly associated with poor OS (HR = 2.06, 95% CI: 1.69–2.52, *P* < 0.01) and PFS (HR = 2.78, 95% CI: 1.69–4.55, *P* < 0.01) in various cancers. Moreover, the promoted *SNHG1* expression was also associated with tumor progression ((III/IV vs. I/II: HR = 1.89, 95% CI: 1.53–2.34, *P* < 0.01). In stratified analyses, a significantly unfavorable association of elevated lncRNA *SNHG1* and OS was observed in both digestive system (HR = 2.04, 95% CI: 1.56–2.68, *P* < 0.01) and non-digestive system (HR = 2.09, 95% CI: 1.55–2.83, *P* < 0.01) cancer patients.

**Conclusions:**

The present analysis indicated that the increased *SNHG1* is associated with poor OS in patients with general tumors and may be served as a useful prognostic biomarker.

**Electronic supplementary material:**

The online version of this article (10.1186/s12885-019-5987-4) contains supplementary material, which is available to authorized users.

## Background

Cancer has gradually becoming a major threaten for human health in the worldwide [1, 2]. Even though tremendous improvements had been made in cancer treatment, the long-term survival rate still remains unsatisfied in various types of cancers. The molecular mechanism underlying oncogenesis and tumor progression is still not fully elucidated, which restrict the prognostic prediction of cancer patients. Thus, it is urgent for us to identify new effective biomarkers for early diagnosis, prognosis prediction and ideal therapeutic target for cancer patients.

As a class of endogenous non coding RNA, long noncoding RNA (lncRNA) has a broad range of molecular and cellular functions, including chromatin modification, gene imprinting, alternative splicing, dosage compensation, nuclear-cytoplasmic trafficking, and inactivation of major tumor suppressor genes etc. [3–5]. Accumulating evidences of dysregulated lncRNAs in various cancers suggested that these greater than 200 nucleotides RNAs may contribute to cancer development and progression [6, 7]. Moreover, dramatic findings had suggested that lncRNAs may participate in a wide range of biological pathways which underlying oncogenesis [8]. Therefore, lncRNAs have attracted considerable attention as a mighty class of modulators and maybe serve as a potential biomarker for cancer patients [9–12].

LncRNA-*SNHG1* (small nucleolar RNA host gene 1), located in 11q12.3, is expressed in broad ranges of cancer tissues [13–16]. Recently, emerging evidence from fundamental and clinical studies revealed that lncRNA-*SNHG1* participates in tumorigenesis and exhibits poor prognostic value in different types of cancers. However, most studies reported the prognostic value of *SNHG1* in cancer patients was limited by small sample size [15, 17]. Therefore, we conducted the present quantitative meta-analysis to investigate the prognostic value of *SNHG1* in various cancers.

## Methods

### Literature search

Articles published in English and Chinese which related to the prognostic value of lncRNA *SNHG1* and tumor progression were eligible for the current analysis. Up to March 20, 2018, a comprehensive search was conducted in several electronic databases: PubMed, Web of Science, Embase, ISI Web of Knowledge, Cochrane Library, BioMed Central, Springer, ScienceDirect, together with three Chinese databases: CNKI, Weipu and Wanfang. Following keywords for the online search in these databases were included: (“long noncoding RNA-” OR “noncoding RNA-” OR “lnc RNA-” OR “small nucleolar RNA host gene 1” OR “*SNHG1*”) AND (“carcinoma” OR “cancer” OR “tumor” OR “neoplasm”) AND (“prognosis” OR “prognostic”). The reference lists of primary publications were also manually searched to achieve potential eligible studies.

### Inclusion and exclusion criteria

The following selection criteria for the eligible studies were used: 1) Definite diagnosis or histopathology confirmed for cancer patients; 2) Studies investigating the prognostic features of lncRNA *SNHG1* in any malignant patients; 3) Enough information for the computation of pooled hazard ratios (HR) and 95% confidence intervals (CI). Exclusion criteria for the articles included: Studies absence of prognostic outcomes; 2) Duplicated publications; 3) Non-human research, correspondences, case reports, letters, review articles and other studies without original data.

### Data extraction and quality assessment

Two authors (BZD and CZZ) carefully reviewed the information such as titles, abstracts, full texts and reference lists of each eligible article independently. The enrolled literatures were then qualified by MOOSE and PRISMA checklists (Additional file 1: Table S1 and Additional file 2: Table S2) [18]. In case that the eligible literatures only provide the data as Kaplan–Meier survival curves, the Enguage Digitizer (Version 4.1) software was used to extract the survival information from the graphical plots as the previously described method [19–21]. Extracted items were discussed and any contradiction was arbitrated by a third investigator (YYZ) to reach a consensus. Furthermore, the necessary elements from the enrolled articles were extracted: first author’s name; publication year; cancer resources; tumor type and stage; total cases; follow-up period; lncRNA *SNHG1* detection method; cut-off values; HRs and corresponding 95% CIs.

### Statistical analysis

The present meta analysis was performed with Stata SE 12.0 (Stata Corporation) and RevMan 5.3 software. The main statistical index, HRs and 95% CIs, was calculated for the aggregation of patient survival and tumor progression results. The heterogeneity between studies was determined by *I*^2^ statistics. The fixed effect model was conducted in the studies with no obvious heterogeneity (*I*^2^ < 50%) [21–23]. Potential publication bias was evaluated by performing Begg’s bias test and funnel plot. *P* value less than 0.05 was considered as statistically significant.

## Results

### Eligible studies

**Fig 1:**
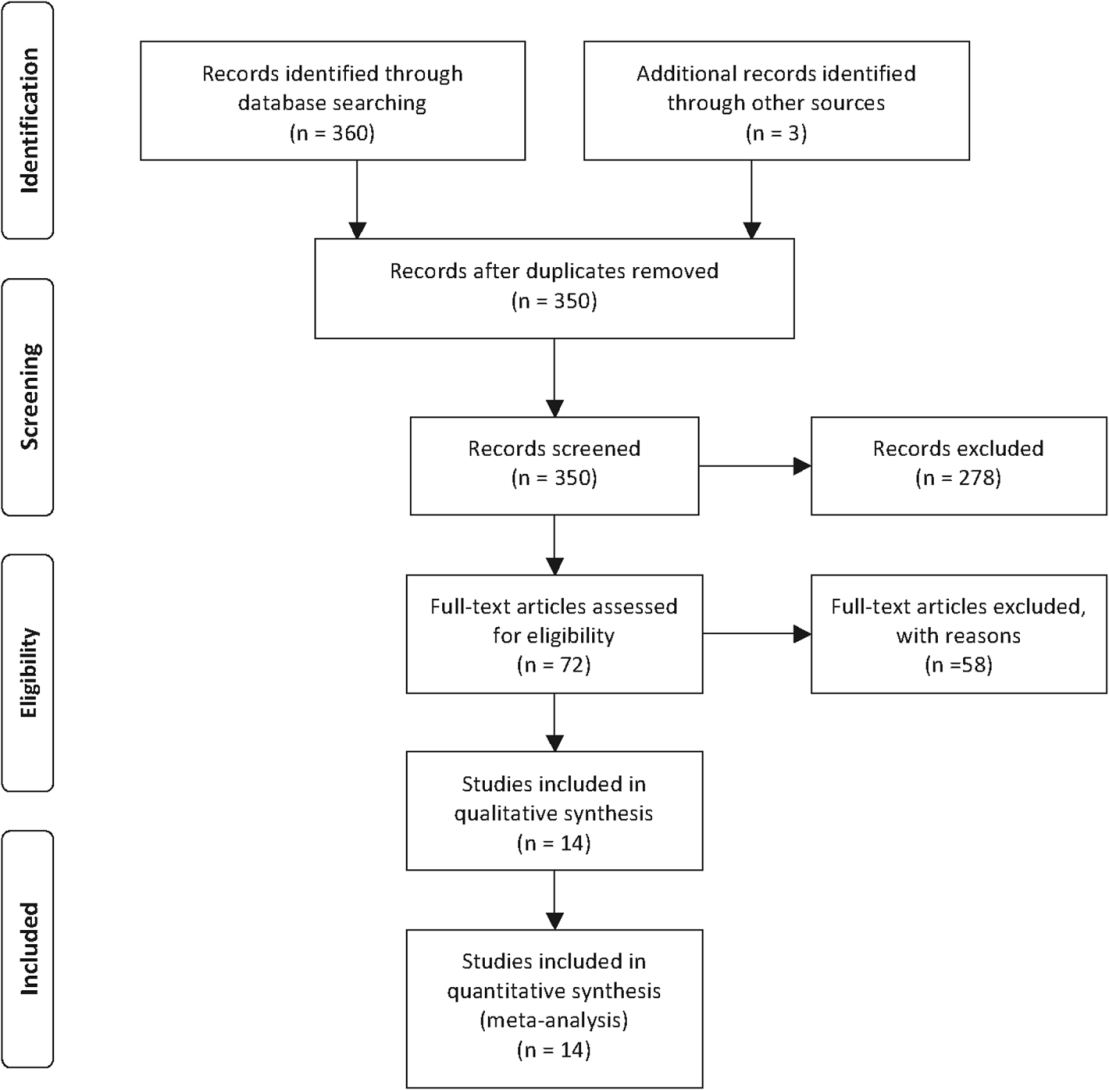
Flow diagram of the study search and selection process

After preliminary online search, 363 literatures in total were originally retrieved from electronic databases. After duplicates removed, 350 potential articles were then subjected to abstract screened. These 278 researches which related to the lncRNA *SNHG1* expression and cancer prognosis were then excluded because they do not match the inclusion criteria. Through carefully full texts assessed the remaining 72 articles, another 58 literatures were then removed according to the exclusion criteria. Ultimately, fourteen articles were enrolled in this present study. The literature screening processes were presented as a flow diagram [14–17, 24–33](Fig. 1).

### Study characteristics

The main features of the enrolled 14 eligible studies included a total of 1397 participants were summarized in Table 1. Briefly, qRT-PCR was used to detect lncRNA *SNHG1* expression level in all of the research studies. The detected cancer tissue samples came from neuroblastoma, esophageal squamous cell cancer, hepatocellular carcinoma, gastric cancer, epithelial ovarian cancer, osteosarcoma, lung squamous cell carcinoma, colorectal cancer, non-small cell lung cancer and lung cancer. Notably, median was selected as cut-off value in different studies. Eight of the fourteen articles focused on the association of *SNHG1* with OS, PFS, EFS or RFS, and two articles investigated both OS and PFS.

**Table 1:** Summary of the 14 included studies

Study	Origin of population	Study design	Disease	N	Stage	Method	Survival analysis	Hazard ratios	Follow-up Months
Divya Sahu 2016	China Taiwan	R	NB	493	IV/I-III	qRT-PCR	OS/EFS	HR/KM	200
Zhang H 2016	China	R	HCC	122	I-II/III-IV	qRT-PCR	NA	NA	NA
Zhang M 2016	China	R	HCC	82	A/B-C	qRT-PCR	OS	K-M	60
Cui 2017	China	R	NSCLC	68	I/II–III	qRT-PCR	OS	KM	60
Hu 2017	China	R	GC	50	NA	qRT-PCR	OS	KM	60
Jiang2017	China	R	OS	25	I-II/III-IV	qRT-PCR	NA	NA	NA
Tang 2017	China	R	LC	43	I-II/III-IV	qRT-PCR	NA	NA	NA
Tian 2018	China	R	CC	82	I-II/III-IV	qRT-PCR	OS/PFS	K-M	120
Wang Q 2017	China	R	Glioma	78	NA	qRT-PCR	OS	NA	60
Wang JD 2018	China	R	OS	45	NA	qRT-PCR	OS	KM	60
Wang Sie 2017	China	R	EOC	67	I-II/III-IV	qRT-PCR	OS	KM	60
Zhang HY 2017	China	R	SCC	62	I-II/III	qRT-PCR	NA	KM	NA
Zhang YJ 2017	China	R	ESCC	72	I- II/III	qRT-PCR	OS	KM	60
Zhu 2017	China	R	CRC	108	I-II/III-IV	qRT-PCR	OS/PFS	HR/KM	60

Study design is described as retrospective (R); *NB* neuroblastoma, *ESCC* esophageal squamous cell cancer, *HCC* Hepatocellular Carcinoma, *GC* gastric cancer, *EOC* epithelial ovarian cancer, *OS* osteosarcoma, *LSCC* Lung squamous cell carcinoma, *CC* colorectal cancer, *NSCLC* non-small cell lung cancer, *LC* Lung cancer

### Meta-analysis

**Fig 2:**
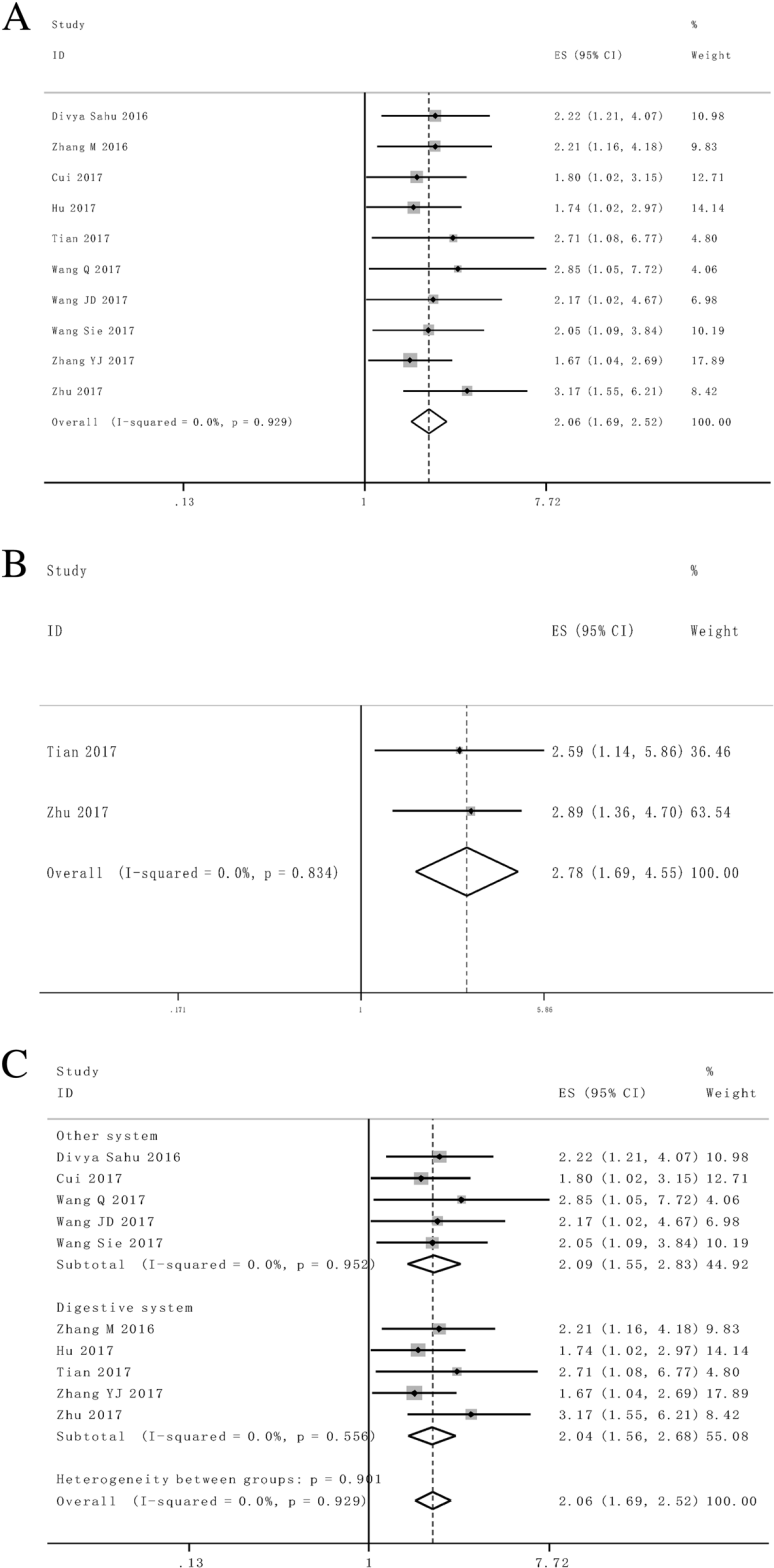
**a** Forest plot for the association between SNHG1 expression levels with overall survival (OS). **b** Forest plot for the association between SNHG1 expression levels with progress free survival (PFS).**c** Stratified analyses for the association between SNHG1 expression with overall survival (OS). Subgroup analysis of HRs of OS by factor of cancer resources

**Fig. 3:**
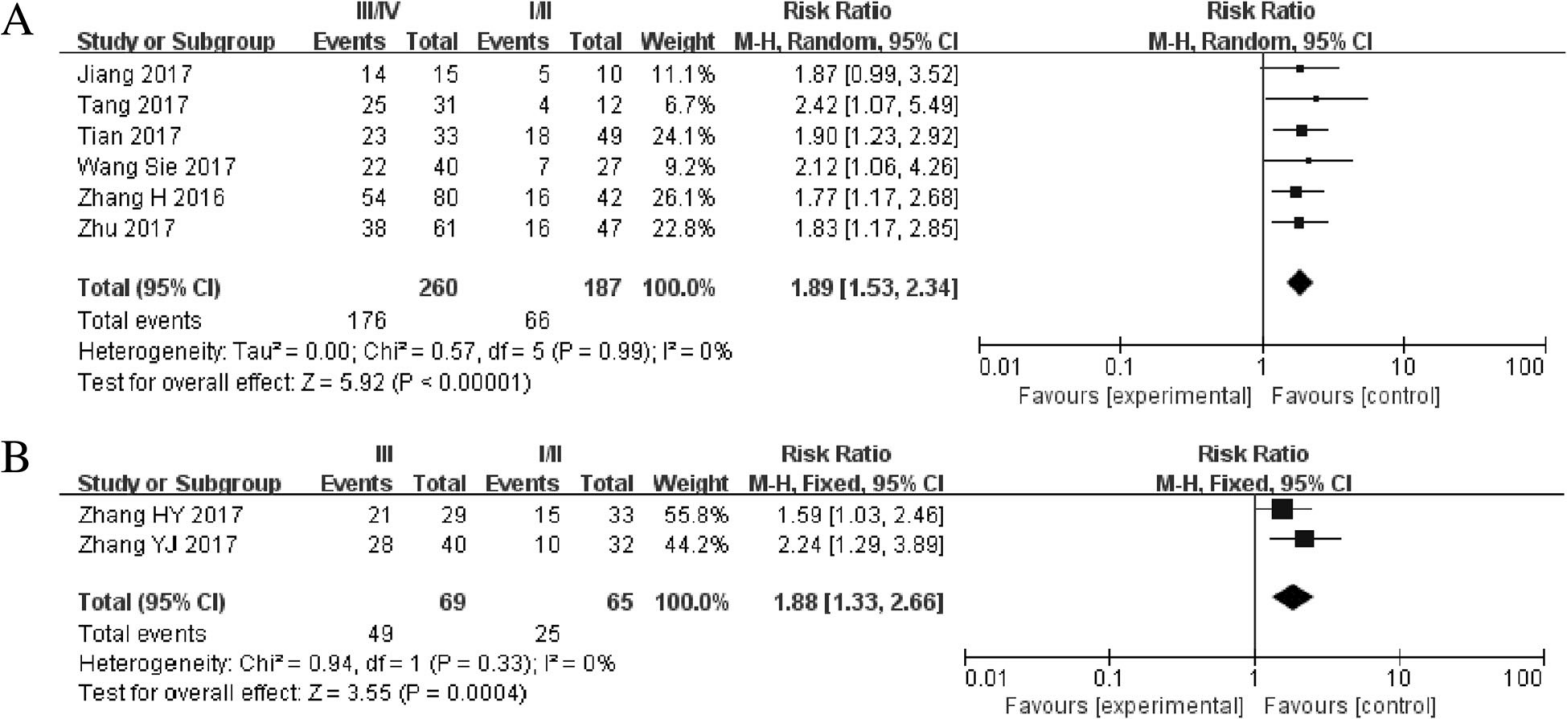
**a** Forest plot for the association between SNHG1 expression with TNM stage (III/IV vs. I/II). **b** Forest plot for the association between SNHG1 expression with TNM stage (III vs. I/II)

Figure 2 presented the forest plot result about lncRNA *SNHG1* and patient outcomes. A fix-effect model was utilized to calculate the pooled effect size because no significant heterogeneity was observed among these enrolled 10 studies (*I*^2^ = 0%). The combined results indicated that the elevated *SNHG1* expression level was significantly predicted poor OS (HR = 2.06, 95% CI: 1.69–2.52, *P* < 0.01) and PFS (HR = 2.78, 95% CI: 1.69–4.55, *P* < 0.01) in various cancers. Moreover, the promoted *SNHG1* level was also associated with tumor progression ((III/IV vs. I/II: HR = 1.89, 95% CI: 1.53–2.34, *P* < 0.01) and (III vs. I/II: HR = 1.88, 95% CI: 1.33–2.66, *P* < 0.01)) (Fig. 3).

### Stratified analysis

Afterwards we set out to throw light upon the prognostic effect of *SNHG1* on different cancer resources. The results from stratified analysis turned out that enforced *SNHG1* expression was predictive of worse outcome in digestive system (HR = 2.04, 95% CI: 1.56–2.68, *P* < 0.01) and non-digestive system (HR = 2.09, 95% CI: 1.55–2.83, *P* < 0.01) cancer patients (Fig. 2c). No significant heterogeneity was found in the subgroup analysis.

### Publication bias

**Fig 4:**
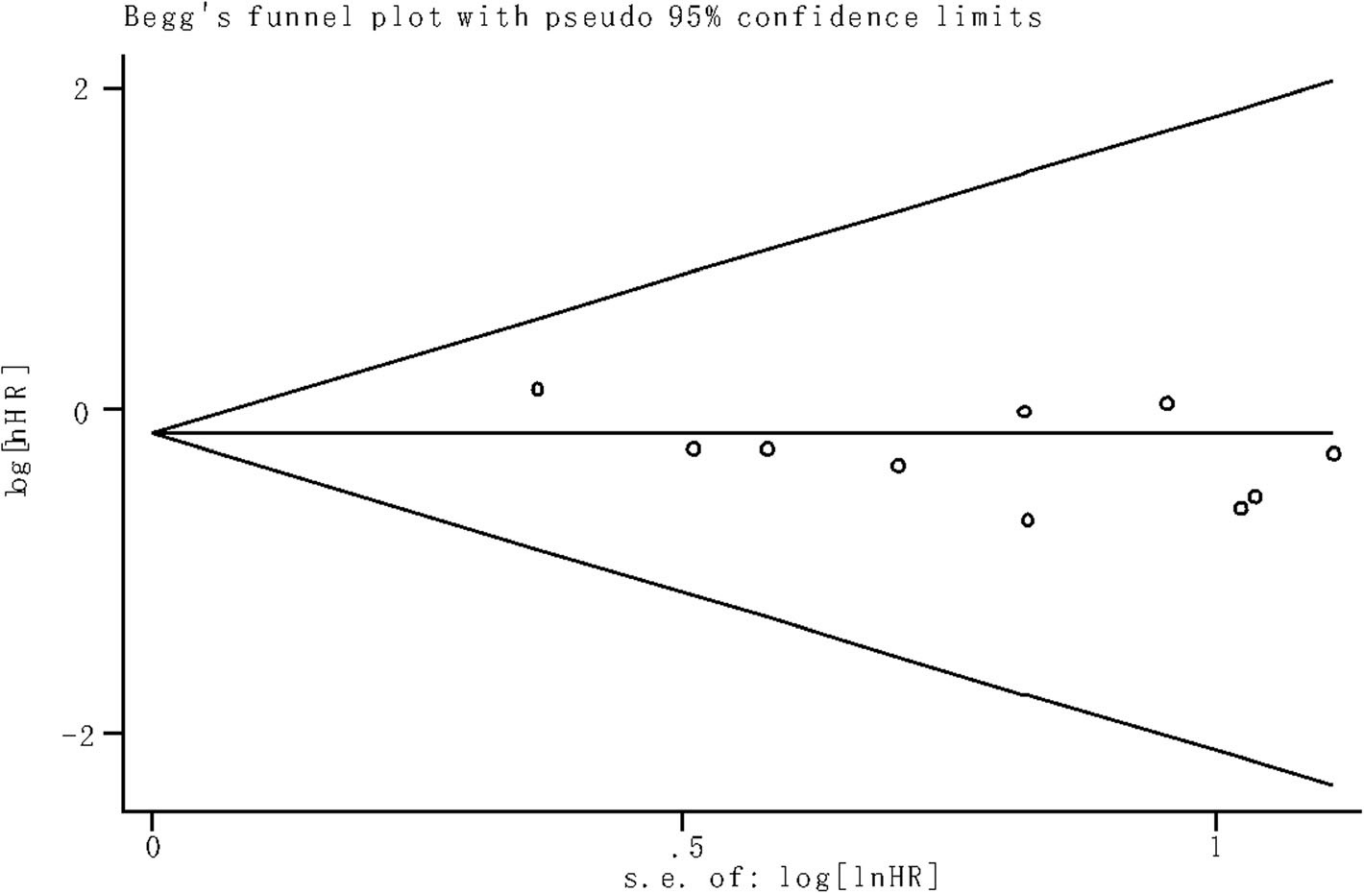
Funnel plot of the publication bias for overall survival

To evaluate publication bias in the current meta-analysis, the indicated studies were conducted with Begg’s bias test and funnel plot analysis. The result of Begg’s test revealed the absence of significant publication bias (*P* = 0.474). The shape of the funnel plot was also symmetrically inverted funnels (Fig. 4).

### Sensitivity analysis

**Fig 5:**
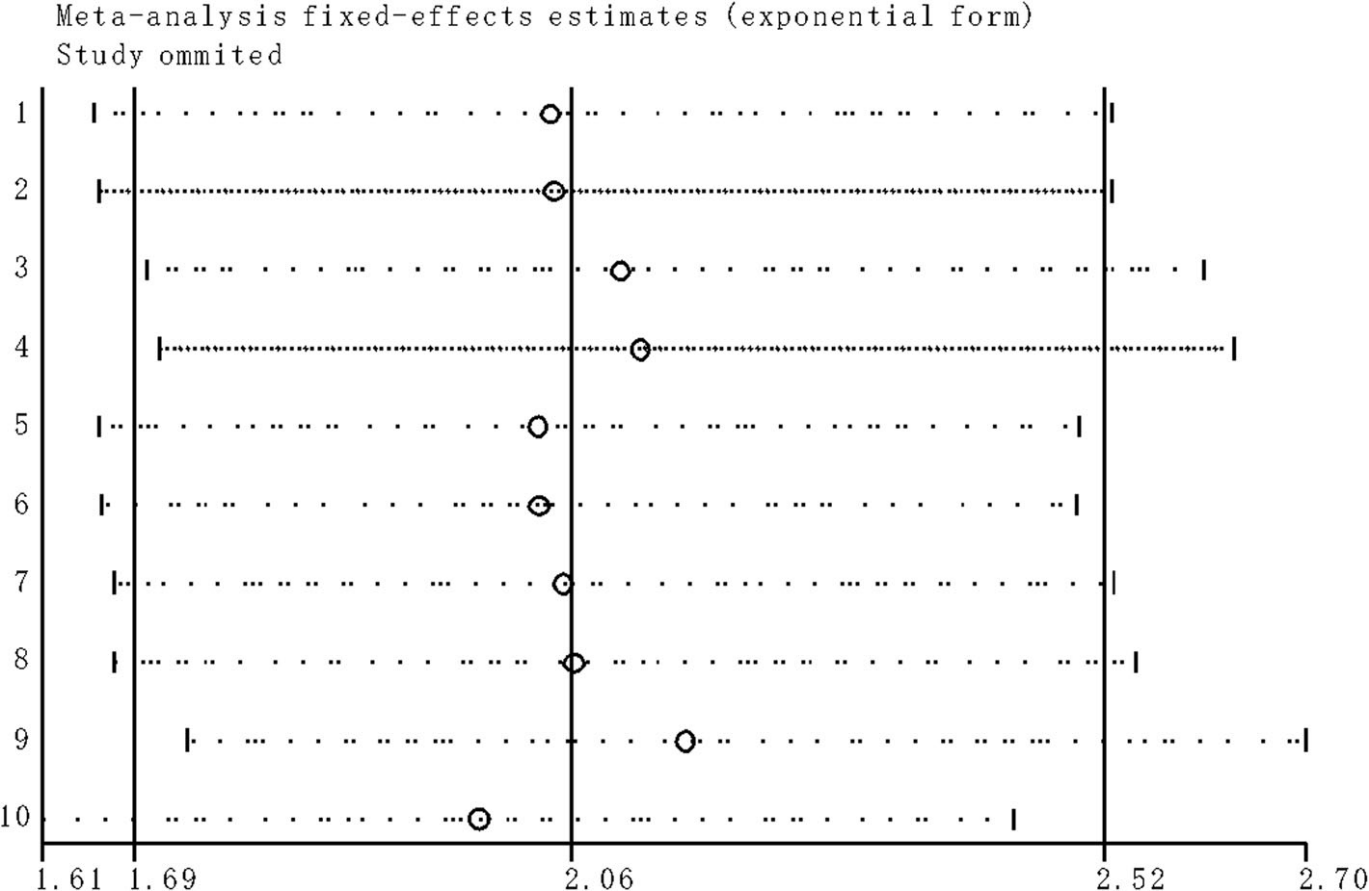
Sensitivity analyses of studies concerning SNHG1 and overall survival

Through sensitivity analysis, it was uncovered that the pooled *SNHG1* HR was not significantly affected by the exclusion of any single study (Fig. 5).

## Discussion

Along with the rapid expanding of high throughput genome sequencing technologies, lncRNAs were demonstrated as novel biomarkers to more precisely evaluate the prognosis of various tumors. Recently, mounting evidence suggested that over-expression lncRNA *SNHG1* correlated with poor prognosis and progression of cancer patients. However, most studies reported the prognostic value of *SNHG1* expression level was limited by small sample size. To the best of our knowledge, there is no systematic meta analysis concerning about lncRNA *SNHG1* expression level and cancer patient outcomes.

LncRNA *SNHG1*, a novel lncRNA located in 11q12.3 region and containing 11 exons, which had been found significantly up-regulated in several types of cancers. The molecular mechanisms prone to be participated in the oncogenesis and progression had gradually been unveiled. For example, the dysregulation of LncRNA *SNHG1* has been demonstrated to participate in Notch and Wnt/β-catenin signaling pathways in osteosarcoma and colorectal cancer [25, 34]. LncRNA *SNHG1* was also suggested to interact with *DNMT1*, *P53* and *TAP63* in GC, HC and LSCC respectively [14, 15, 28]. Moreover, lncRNA *SNHG1* may conduct as a competing endogenous RNA (ceRNA) that exacerbated cancer development. Up-regulated lncRNA *SNHG1* reduces the miRNAs expression level, such as miR-101-3p, miR-145, miR-195, miR-326 and miR-577 in nucleus pulposus cell proliferation, osteosarcoma, hepatocellular carcinoma, non-small cell lung cancer and nasopharyngeal carcinoma respectively [17, 26, 30, 35, 36]. These encouraged evidences urged us investigating the relationship between lncRNA *SNHG1* and cancer prognosis, and our analysis firstly demonstrated that high expression level of lncRNA *SNHG1* was an unfavorable predictor for the clinical outcomes of various cancer patients.

Fourteen online searched studies including 1397 patients in total were pooled in this analysis, which was considered as powerful enough to consolidate our results. Several kinds of tumors, such as neuroblastoma, esophageal squamous cell cancer, hepatocellular carcinoma, gastric cancer, epithelial ovarian cancer, osteosarcoma, lung squamous cell carcinoma, colorectal cancer, non-small cell lung cancer and lung cancer, were implemented in our study. The analysis showed a pooled HR was 2.06 (95% CI: 1.69–2.52, *P* < 0.01), 2.78 (95% CI: 1.69–4.55, *P* < 0.01) and 1.89 (95% CI: 1.53–2.34, *P* < 0.01) for OS, PFS and tumor progression respectively. We also demonstrated that enforced *SNHG1* expression was a predictor of worse outcome in digestive system (HR = 2.04, 95% CI: 1.56–2.68, *P* < 0.01) and non-digestive system (HR = 2.09, 95% CI: 1.55–2.83, *P* < 0.01) cancer patients.

Nevertheless, limitations should be refined when interpreted lncRNA *SNHG1* expression level for cancer outcomes. To start with, although no publication bias was detected by statistical methods, potential bias might exist. Articles with ideal results might be published easily, which might lead to the lack of statistical power. Furthermore, the ethnicity of the cancer patients was Asian and our results may best elucidate the correlation of lncRNA *SNHG1* with Asian patients.

In summary, despite some limitations mentioned above, our meta-analysis indicated that the elevated lncRNA *SNHG1* level is significantly associated with cancer patients’outcome. To strengthen our results, well-designed clinical studies and multi-ethnics clinical researches should be carried out before lncRNA *SNHG1* could be applied as a prognostic marker in the routine clinical guidance of cancer patients.

## Conclusions

In conclusion, the present results suggest that promoted lncRNA *SNHG1* expression levels are associated with OS and lncRNA *SNHG1* may be used as a prognostic marker for cancer patients.

## Additional files

Additional file 1: MOOSE checklist. (DOCX 15 kb)

Additional file 2: PRISMA checklist. (DOCX 18 kb)

## Data Availability

All data analyzed during this study are included in this published article.

## Abbreviations

95% CI: 95% confidence interval
ceRNA: Competing endogenous RNA
EFS: Event free survival
HR: Hazard ratio
LncRNA: Long noncoding RNA
OS: Overall survival
PFS: Progress free survival
RFS: Relapse free survival
*SNHG1*: Small nucleolar RNA host gene 1
